# Pain Status and Its Association with Physical Activity, Psychological Stress, and Telework among Japanese Workers with Pain during the COVID-19 Pandemic

**DOI:** 10.3390/ijerph18115595

**Published:** 2021-05-24

**Authors:** Takahiko Yoshimoto, Tomoko Fujii, Hiroyuki Oka, Satoshi Kasahara, Kayo Kawamata, Ko Matsudaira

**Affiliations:** 1 Department of Hygiene, Public Health and Preventive Medicine, Showa University School of Medicine, Tokyo 142-8555, Japan; 2 Department of Medical Research and Management for Musculoskeletal Pain, 22nd Century Medical & Research Center, Faculty of Medicine, The University of Tokyo, Tokyo 113-8655, Japan; ort4771@gmail.com (T.F.); okah-tky@umin.ac.jp (H.O.); cairns2003@hotmail.com (K.K.); kohart801@gmail.com (K.M.); 3 Department of Anesthesiology and Pain Relief Center, The University of Tokyo Hospital, Tokyo 113-8655, Japan; namahage@king.odn.ne.jp

**Keywords:** pain, telework, physical activity, psychological stress, COVID-19

## Abstract

Changes in working styles and physical activities, and an increase in psychological stress during the coronavirus disease 2019 (COVID-19) pandemic, may have affected pain conditions among workers with pain; however, these associations are still poorly understood. Therefore, we conducted a web-based, cross-sectional study to investigate these changes among Japanese workers suffering from pain. A total of 1941 workers who were aged 20–64 years and suffered from body pain within 4 weeks prior to the study were included. Information was collected using a self-reported questionnaire between July and August 2020. Among the respondents, 15% reported that their pain worsened during the COVID-19 pandemic. Approximately half of the workers claimed to have decreased physical activity (47%) and increased psychological stress (47%) during the pandemic. Multivariable logistic regression analyses found that telework (odds ratio 2.27, 95% confidence interval 1.68–3.06), decreased physical activity (3.18, 2.38–4.27), and increased psychological stress (2.16, 1.64–2.84) were associated significantly with pain augmentation. The group of workers who participated in telework and had decreased physical activity comprised the highest proportion of those with augmented pain. Our findings suggest that measures, which consider physical activities, psychological aspects, and working styles, to alleviate pain may be required for the working population in the future.

## 1. Introduction

Since the beginning of 2020, the novel coronavirus disease 2019 (COVID-19) has spread rapidly worldwide. In many countries, various measures that have centered on social distancing have been implemented to control the spread of the infection [1,2]. In Japan, on 7 April 2020, due to the widespread occurrence of COVID-19 in the country, the Japanese government declared a state of emergency in seven prefectures, including the capital area, and then nationwide [3]. To reduce social contact with others, citizens were required to refrain from going out unless necessary. This emergency strategy imposed significant changes in working environments, such as the implementation of telework, as well as in the lifestyles of the workers.

Globally, pain is a common health problem that could result in a substantial economic burden [4,5]. Previously, we conducted a large-scale survey on health conditions and work productivity, and reported that pain symptoms such as neck pain, low back pain, or headache were the leading causes of reduced work productivity [6]. The negative impact of the COVID-19 pandemic on workers suffering from pain is of concern [7]. Recently, several studies have reported that many people with chronic pain or low back pain have experienced a worsening of their pain during the COVID-19 pandemic [8,9,10]. It is important to understand the situation of workers who experienced pain during the pandemic in order to employ appropriate pain management modalities for such patients in the future, after the COVID-19 pandemic.

Due to the declaration of the state of emergency, many companies were requested to implement working styles such as telework to avoid personal contact. While the introduction of telework has many advantages including flexibility in time and/or space, and work–life balance [11], there are also concerns that this type of working style may have negative health effects, such as musculoskeletal pain. Previous studies have reported that neck pain or low back pain became worse among workers who performed telework or distance learning during the COVID-19 pandemic [9,12]. In addition to the change in working style, the government also encouraged people to refrain from going out unless necessary, which could also have led to a decrease in physical activity [13,14] and an increase in psychological stress [15,16]. Many studies have indicated that low levels of physical activity and the presence of psychological problems were significantly associated with pain [17,18,19]. These changes in working styles and physical activities, and the increase in psychological stress during the COVID-19 pandemic, may affect pain conditions among workers with pain; however, these associations are still poorly understood.

The aim of this study was to investigate the impact of the COVID-19 pandemic on pain conditions, physical activities, psychological stress, and working styles among Japanese workers suffering from pain, and to examine the factors associated with pain augmentation.

## 2. Materials and Methods

### 2.1. Study Population

**Figure 1:**
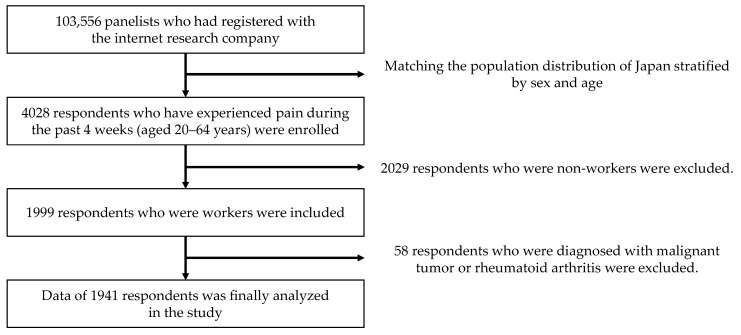
Flowchart of the sampling procedure for the analysis.

We conducted a cross-sectional, web-based survey on pain among Japanese adults between 29 July 2020 and 19 August 2020 (the period of the second wave in Japan). Participants were recruited from an internet research agency, hamon Co., Ltd. (Kanagawa, Japan) which had approximately 1.58 million panelists who had voluntarily registered. A questionnaire was distributed via e-mail to 103,556 panelists stratified by sex and age (Figure 1). The questionnaire was displayed to panelists who consented to participate in the study. The questionnaire was designed to be rejected automatically if there were missing data or incomplete responses. The inclusion criteria were (1) aged 20–64 years, and (2) have experienced pain anywhere in their body during the past 4 weeks. The survey was closed when the number of respondents reached 4028, which was determined roughly according to the population distribution (sex and age) in Japan [20]. Of these respondents, we selected the data of workers for the analyses. Workers included part time job workers, temporary workers, and freelance workers as well as regular workers. Individuals who were unemployed, retired, students who were not working, or full-time homemakers were excluded from the study. We further excluded participants who were diagnosed with malignant tumor or rheumatoid arthritis. All protocols in the study were approved by the Institutional Review Board of The University of Tokyo (No. 2019296NI).

### 2.2. Measurements

Pain: Participants were asked about their pain with the following question: how did the declared state of emergency or self-restrictions on going out due to the COVID-19 pandemic affect your pain? The response categories were as follows: (1) the pain worsened considerably, (2) pain worsened somewhat, (3) there was no change in the pain, (4) pain improved somewhat, (5) pain improved considerably or almost disappeared. The responses (1) and (2) were defined as pain augmentation, and the respondents who answers (1) or (2) were categorized as the “pain augmentation group”. Then, they were asked to identify the body regions in which pain worsened, with multiple answers allowed. The questionnaire included an illustration of a mannequin with the body regions illustrated (head, neck, upper back, shoulder, elbow, wrist/hand, lower back/buttock, thigh, knee, ankle/foot, and others).

Working style: Participants were asked whether the opportunities for telework increased with the declared state of emergency or self-restrictions on going out due to the COVID-19 pandemic. The response categories were as follows: (1) My job cannot adopt a telework style, (2) telework has not been introduced although it is possible to work remotely in my job, (3) I started teleworking during the COVID-19 pandemic, (4) telework was introduced before the pandemic and the frequency of telework has not changed, (5) telework was introduced before the pandemic and the frequency of telework has increased, (6) telework was introduced before the pandemic and the frequency of telework has decreased, (7) I am not currently working (including leave from job). Respondents who answered (3) or (5) were defined as ‘started/increased’ telework, and those who answered the other options were defined as ‘not started/increased’ telework.

Physical activity and psychological stress: Change in physical activity since the start of the COVID-19 pandemic was evaluated using the following question: how has the COVID-19 pandemic impacted the amount of exercise/physical activity you participate in (including the time taken to walk when commuting, housework, or care giving), with the self-restriction on going out, changes in working styles, or changes in your family’s lifestyles? Respondents were evaluated on a 5-point categorical scale (decreased considerably, decreased somewhat, no change, increased somewhat, increased considerably). Decreased physical activity was defined as the responses of decreased somewhat or considerably. In addition, participants were asked about the influences of the COVID-19 pandemic on psychological stress using the following question: how has the COVID-19 pandemic impacted your stress levels with the declared state of emergency or the self-restrictions on going out? Response options were the same as for physical activity. Responses of increased somewhat or considerably were defined as an increase in psychological stress.

Other variables: Information on sex, age, body height, weight, marital status, education level, employment status, and industry type was also collected through the self-reported questionnaire. The response categories were as follows: marital status (married, unmarried, divorced, widowed), education level (middle school, high school, vocational school, higher professional school, college, graduate college, other), and employment status (regular employee, part-time worker, temporary worker, business executive, family business worker, home worker, student, homemaker, inoccupation, or other). Industry type was obtained using the Japanese Standard Industrial Classification [21], and these types were classified into the following groups: primary (agriculture, forestry, and fishing), secondary (mining, construction, and manufacturing), tertiary industries (mainly white-collar types), and others (unable to classify). These industry types corresponded roughly to the International Standard Industrial Classification. Body mass index (BMI) was calculated by dividing the body weight by height squared.

### 2.3. Statistical Analyses

We checked the normality of data distribution using the Anderson–Darling test, and found that none of the data analyzed in the study were normally distributed; therefore, all continuous variables were presented as medians (25%, 75% percentiles). The characteristics of the study participants were compared between the pain augmented and not-augmented groups using the Wilcoxon rank-sum test for continuous variables and the chi-square test or Fisher’s exact test for categorical variables. To examine the association of pain augmentation with changes of several conditions during the COVID-19 pandemic, a logistic regression model was used to calculate the odds ratio (OR) and the 95% confidence interval (CI). We adjusted for sex, age, BMI, marital status, education level, employment type, industry type, and other explanatory variables as potential confounders in the model. Multicollinearity was not suspected, as all the variance inflation factors were <0.2. Using a multivariable logistic regression analysis, we further examined the association between pain augmentation and a combination of telework with physical activity. A two-sided *p*-value < 0.05 was considered to be statistically significant. All the statistical analyses were performed using SAS version 9.4 (SAS Institute, Inc., Cary, NC, USA).

## 3. Results

**Figure 2:**
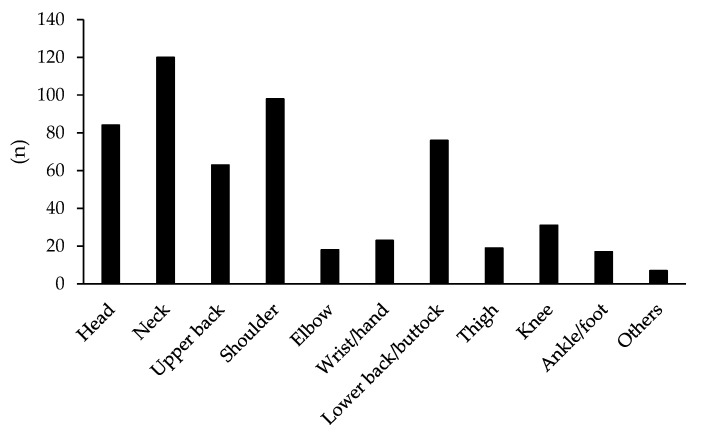
Body parts where pain was augmented during the declared state of emergency or while refraining from going outside during the COVID-19 pandemic.

Among the 4028 respondents who experienced pain somewhere in their body during a past 4 weeks, 1941 workers were analyzed in our study. Table 1 shows the characteristics of these workers. The median age (25, 75 percentiles) of the included participants were 43 (33, 52) years, and 70.5% of the participants were men. With regard to changes in pain with the spread of COVID-19, 282 workers (14.5%) had worsened pain, 74.5% had no change in pain, and 10.9% had decreased pain. The main body regions where pain worsened were the neck, shoulder, head, and lower back/buttocks (Figure 2). Of all the included participants, 521 workers (26.8%) started teleworking or increased their frequency of teleworking. Since the beginning of the COVID-19 pandemic, approximately half of the workers (47.2%) answered that their physical activity decreased, while psychological stress increased in 908 workers (46.8%). Table 1 also shows the comparison of the characteristics between workers with and without pain augmentation. The proportion of women in workers with pain augmentation was higher than that in those without pain augmentation. Workers with pain augmentation were younger than those without pain augmentation. The proportions of those with decreased physical activity, increased psychological stress, and having started/increased telework were significantly higher in workers with pain augmentation than in those without pain augmentation (*p* < 0.001 in all).

The association of pain augmentation with telework, physical activity, and psychological stress since the start of the COVID-19 pandemic is shown in Table 2. A logistic regression analysis indicated that started/increased telework (OR 2.32: 95%CI 1.79–3.02), decreased physical activity (OR 3.78: 95%CI 2.85–5.01), and increased psychological stress (OR 2.22: 95%CI 1.71–2.88) were associated significantly with pain augmentation. The results remained significant after adjusting for several confounders.

We further analyzed the association between ‘the combination of telework condition and change in physical activity’ and pain augmentation. The adjusted OR (95%CI) for pain augmentation was 3.18 (1.88–5.36) in ‘started/increased telework plus not decreased physical activity’, 3.74 (2.60–5.38) in ‘not started/increased telework plus decreased physical activity’, and 7.45 (4.97–11.18) in ‘started/increased telework plus decreased physical activity’ (Table 3).

## 4. Discussion

In the present study, we investigated the impact of the COVID-19 pandemic on pain, physical activity, stress, and working style among Japanese workers with pain. In addition, we analyzed the factors associated with pain augmentation. Our results show that pain worsened during the COVID-19 pandemic in 15% of workers. Multivariable analyses found that telework, decreased physical activity, and increased psychological stress were associated significantly with pain augmentation. In addition, workers who participated in telework and experienced a decrease in physical activity comprised the highest proportion of those with augmented pain. Our findings indicate that physical activity, psychological aspects, and telework may need to be considered in pain management among workers during the COVID-19 pandemic.

### 4.1. Telework and Pain

Among the included participants, approximately one in four workers commenced telework or increased their amount of telework during COVID-19 pandemic. It can be considered that many companies were semi-forced to suddenly change their ways of working due to the spread of COVID-19, which may have resulted in inadequate work environments such as small workspaces with furniture that was unsuitable for work. A survey in Spain that examined the characteristics of remote workers since the beginning of the COVID-19 pandemic reported that the home environment, including the work equipment, was not adequate [12]. Working while sitting on sofas or working for long hours on a notebook personal computer (PC) with a relatively small screen is likely to cause/exacerbate musculoskeletal pain. The use of mobile devices including laptop/notebooks or tablets, commonly used by teleworkers, can lead to poor posture, such as neck flexion, compared with the use of desktop PCs [22,23]. Several studies have indicated that sitting with a poor posture can increase the risk of musculoskeletal disorders such as neck or low back pain [24,25]. Indeed, we found that the main body areas where the pain worsened were the neck, shoulder, and lower back in the present study. In a previous study with mobile device usage postures, it was indicated that working with a mobile device on one’s lap without a desk affected the development of musculoskeletal disorders [26]. Although our study did not obtain information on the work environment among teleworkers, in promoting telework in the future, the perspective of occupational health management including work environment will be required to reduce painful conditions among workers. Companies may need to provide financial support to purchase telework-adapted equipment [27] while utilizing the subsidies established by several ministries and local governments in Japan to prepare optimal teleworking environments.

### 4.2. Physical Activity and Pain

In our study, the proportion of workers with decreased physical activity was significantly higher in the pain augmentation group (73.8%) than in the group without pain augmentation (42.7%). Physical disuse caused by a decrease in physical activity has been considered to be involved in the chronicity of pain [28]. A previous study demonstrated that workplace physical activity interventions had the potential to reduce musculoskeletal pain [29]. Moreover, a recent systematic review on the association between physical activity and low back pain found that physical activity was associated significantly with a decreased risk of low back pain [17]. These studies have highlighted the important role of physical activity in pain management. In our study population, a decrease in physical activity during the COVID-19 pandemic may have had a negative effect on pain conditions among workers. Further prospective or interventional studies are needed to verify the causal relationship.

The working style of telework can lead to a decrease in physical activity [30], with reductions in movement within the company or the opportunity for commuting. We therefore conducted a sub-analysis by classifying workers into four groups with a combination of telework and change in physical activity. Our results reveal that among the four groups, in the group of ‘started/increased telework’ plus ‘decreased physical activity’, the proportion of workers with pain augmentation was the highest. Bouziri et al. proposed that the preparation of the home environment to make it suitable for telework and promotion of physical activities were key measures to reduce occupational health risks such as musculoskeletal pain while working at home in the era of COVID-19 [27]. Our results suggest that measures to maintain/increase usual physical activity may be added to the approach to manage pain conditions in teleworkers.

### 4.3. Psychological Stress and Pain

Our results indicate that an increase in psychological stress since the COVID-19 pandemic was also associated with pain augmentation among workers suffering from pain. The common types of psychological stress included a fear of contracting COVID-19, changes in lifestyles, financial problems, and problems with one’s own health. Rogers et al. indicated that, compared to individuals without pain, those with pain reported significantly higher levels of psychological problems while COVID-19 fear and sleep problems were associated with pain intensity [31]. Psychological stress could have led to anxiety or depressive conditions, which may have been involved in pain augmentation. Psychological aspects have been recognized as an important factor in the chronicity of pain from the perspective of a biopsychosocial model [19]. Given that it is well-recognized that pain and psychological problems have a bidirectional relationship, further research is needed to examine the direction of the relationship between the two found in our study.

Our study has several limitations that should be considered. First, the study participants may not be truly representative of the general population in Japan. The sample analyzed in this study included a higher percentage of men (70%) than that in the national statistical data of Japanese workers. In addition, the recruitment of participants in web-based surveys, such as ours, causes concern regarding sampling issues, which have been described previously [32]. Individuals without Internet access could not participate in our survey. Moreover, only workers who were interested in their own health may have tended to respond to our survey. This limited the generalizability of our results. Second, we used unvalidated questionnaires on changes in pain, physical activity, and stress. Moreover, a retrospective assessment of subjective changes in some conditions before and after the pandemic may be associated with recall bias. Hence, further studies using validated questionnaires are needed to verify our findings. Third, the characteristics of companies which introduced telework could not be adequately considered. A company which can introduce the style of telework may be large-scale and occupation-specific, such as those with white collar or desk workers. We performed a sensitivity analysis by limiting the participants in industries with a high percentage of those who carried out teleworking or increased the frequency of telework (information and communications, finance, insurance, real estate, research, education, and manufacturing) (Table S1). The analysis rendered results similar to our main results (Table S2). Fourth, our study could not verify the direction of association because of the cross-sectional nature of the study design. For example, it cannot be ruled out that a decrease in physical activity may have resulted from pain augmentation. We expect our study to trigger discussions on pain management in workers. Lastly, the effects of a lack of access to clinics/facilities for pain treatment/management due to the COVID-19 pandemic [33] or general health status were not considered in our analysis, as we did not collect this information in the questionnaire.

## 5. Conclusions

We found that 15% of workers with pain experienced the augmentation of their pain during the COVID-19 pandemic. Teleworking, physical activity, and psychological stress were associated significantly with pain augmentation. Moreover, the proportion of workers whose pain worsened was highest in the group of ‘started/increased telework plus decreased physical activity’. Our findings obtained from the unprecedented situation by the COVID-19 pandemic suggest that pain management considering physical activities, psychological aspects, and working styles may be required in workers with pain.

## Figures and Tables

**Table 1:** Characteristics of the study participants.

	All	Pain		
		Augmented	Not Augmented	*p*-Value
	(*N* = 1941)	(*n* = 282)	(*n* = 1659)	
Sex, n (%)				
Men	1368 (70.5)	182 (64.5)	1186 (71.5)	0.018
Women	573 (29.5)	100 (35.5)	473 (28.5)	
Age, years	43 (33, 52)	40 (30, 48)	43 (33, 52)	<0.001
BMI, kg/m^2^	22.1 (19.9, 24.7)	21.6 (19.7, 24.4)	22.2 (20.0, 24.7)	0.143
Marital status, n (%)				
Married	962 (49.6)	135 (47.9)	827 (49.8)	0.677
Unmarried	850 (43.8)	130 (46.1)	720 (43.4)	
Divorced or widowed	129 (6.7)	17 (6.0)	112 (6.8)	
Education level, n (%)				
No college	813 (41.9)	107 (37.9)	706 (42.6)	0.147
College	1128 (58.1)	175 (62.1)	953 (57.4)	
Employment type, n (%)				
Regular	1167 (60.1)	181 (64.2)	986 (59.4)	0.132
Non-regular	774 (39.9)	101 (35.8)	673 (40.6)	
Industry type, n (%)				
Primary industry	21 (1.1)	5 (1.8)	16 (1.0)	0.444
Secondary industry	463 (23.9)	64 (22.7)	399 (24.1)	
Tertiary industry	1328 (68.4)	191 (67.7)	1137 (68.5)	
Others	129 (6.6)	22 (7.8)	107 (6.4)	
Conditions during COVID-19 pandemic				
Physical activity, n (%)				
Decreased	916 (47.2)	208 (73.8)	708 (42.7)	<0.001
Not decreased	1025 (52.8)	74 (26.2)	951 (57.3)	
Psychological stress, n (%)				
Increased	908 (46.8)	179 (63.5)	729 (43.9)	<0.001
Not increased	1033 (53.2)	103 (36.5)	930 (56.1)	
Teleworking, n (%)				
Started/increased	521 (26.8)	120 (42.6)	401 (24.2)	<0.001
Not started/increased	1420 (73.2)	162 (57.4)	1258 (75.8)	

BMI, body mass index; COVID, coronavirus disease. Values are presented as median (25, 75 percentiles), except where indicated as n (%).

**Table 2:** Association of pain augmentation with telework, physical activity, and psychological stress since the start of the COVID-19 pandemic.

		Pain Augmented	Crude		Adjusted *	
		*n* (%)	OR	95%CI	OR	95%CI
Telework	Started/increased	120 (23.0)	2.32	1.79–3.02	2.27	1.68–3.06
	Not started/increased	162 (11.4)	1.00		1.00	
Physical activity	Decreased	208 (22.7)	3.78	2.85–5.01	3.18	2.38–4.27
	Not decreased	74 (7.2)	1.00		1.00	
Psychological stress	Increased	179 (19.7)	2.22	1.71–2.88	2.16	1.64–2.84
	Not increased	103 (10.0)	1.00		1.00	

OR, odds ratio; CI, confidence interval. * Adjusted for sex, age, body mass index, marital status, education level, employment type, industry type, telework, physical activity, psychological stress.

**Table 3:** Association between ‘the combination of telework and physical activity’ and pain augmentation.

		Pain Augmented	Crude		Adjusted *	
	*N*	*n* (%)	OR	95%CI	OR	95%CI
Telework (+) and PA decreased (+)	326	93 (28.5)	6.65	4.55–9.72	7.45	4.97–11.18
Telework (−) and PA decreased (+)	590	115 (19.5)	4.03	2.82–5.77	3.74	2.60–5.38
Telework (+) and PA decreased (−)	195	27 (13.8)	2.68	1.62–4.42	3.18	1.88–5.36
Telework (−) and PA decreased (−)	830	47 (5.7)	1.00		1.00	

PA, physical activity; OR, odds ratio; CI, confidence interval. * Adjusted for sex, age, body mass index, marital status, education level, employment type, industry type, psychological stress.

## Data Availability

The data presented in this study are available from the corresponding author on reasonable request.

## Supplementary Materials

The following are available online at https://www.mdpi.com/article/10.3390/ijerph18115595/s1, Table S1: Details of the industry type of study participants, Table S2: Association of pain augmentation with telework, physical activity, and psychological stress among workers in industries with a high rate of teleworking (*n* = 749).
