# Supplementary material for: Pain Status and Its Association with Physical Activity, Psychological Stress, and Telework among Japanese Workers with Pain during the COVID-19 Pandemic
Source: Int J Environ Res Public Health. 2021 May 24;18(11):5595. doi: 10.3390/ijerph18115595 (PMC8197253; doi:10.3390/ijerph18115595)
Supplement: Supplementary file 1 [file ijerph-18-05595-s001.zip › ijerph-1208054-supplementary.pdf]

Supplementary Material: Table S1

Table S1. Details of the industry type of study participants.

Industrial Classification	All	Telework Started/Increased	
		n	%
Agriculture, forestry	18	3	16.7
Fishery	3	1	33.3
Mining and quarrying of stone and gravel	0	0	0.0
Construction	85	11	12.9
Manufacturing	378	129	34.1
Electricity, Gas, Heat supply and Water	23	5	21.7
Information and communications	101	69	68.3
Transport and postal activities	100	15	15.0
Wholesale and Retail trade	199	42	21.1
Finance and Insurance	63	28	44.4
Real estate and goods rental and leasing	49	18	36.7
Scientific research, professional and technical services	57	20	35.1
Accommodations, eating and drinking services	75	7	9.3
Living-related and personal services and amusement services	59	9	15.3
Education, learning support	101	37	36.6
Medical, health care and welfare	169	9	5.3
Compound services	22	7	31.8
Services, n.e.c	241	67	27.8
Government services	69	23	33.3
Others (unable to classify)	129	21	16.3

Supplementary Material: Table S2

Table S2. Association of pain augmentation with telework, physical activity, and psychological stress among workers in industries with a high rate of teleworking (n = 749).

		Pain Augmented	Crude		Adjusted *	
		n (%)	OR	95%CI	OR	95%CI
Telework	Started/increased	66 (21.9)	2.40	1.59–3.60	2.22	1.40–3.53
	Not started/increased	47 (10.5)	1.00		1.00	
Physical activity	Decreased	81 (21.2)	2.80	1.81–4.34	2.44	1.55–3.85
	Not decreased	32 (8.7)	1.00		1.00	
Psychological stress	Increased	64 (18.6)	1.65	1.10–2.47	1.67	1.09–2.54
	Not increased	49 (12.1)	1.00		1.00	

OR, odds ratio; CI, confidence interval. * Adjusted for sex, age, body mass index, marital status, education level, employment type, industry type, telework, physical activity, psychological stress.
